# Neuroprotective Effects of Shenqi Fuzheng Injection in a Transgenic SOD1-G93A Mouse Model of Amyotrophic Lateral Sclerosis

**DOI:** 10.3389/fphar.2021.701886

**Published:** 2021-10-19

**Authors:** Kazuo Sugimoto, Jia Liu, MingXuan Li, YueBo Song, Chi Zhang, ZhiGuang Zhai, Ying Gao

**Affiliations:** ^1^ Department of Neurology, Dongzhimen Hospital, Beijing University of Chinese Medicine, Beijing, China; ^2^ Institute for Brain Disorders, Beijing University of Chinese Medicine, Beijing, China; ^3^ Institute of Basic Theory for Chinese Medicine, China Academy of Chinese Medical Sciences, Beijing, China; ^4^ Beijing University of Chinese Medicine Third Affiliated Hospital, Beijing, China

**Keywords:** Shenqi Fuzheng Injection, amyotrophic lateral sclerosis, neurodegeneration, neuroprotective effect, oxidative stress, Nrf2 pathway

## Abstract

**Background:** Amyotrophic lateral sclerosis (ALS) is a progressive neurodegenerative disease, in the pathogenesis of which oxidative stress (OS) was believed to play a key role. Shenqi Fuzheng Injection (SFI) concocted from two kinds of Chinese medicinal herbs, Radix Codonopsis and Radix Astragali, was proven to be eligible to reduce the OS injury and increase the activity of the nuclear factor-erythroid-2–related factor 2 (Nrf2) pathway, an antioxidant enzymes inducer.

**Objective:** We aim to investigate the effects and potential mechanisms underlying the action of SFI on a well-established transgenic mouse model of ALS.

**Methods:** Transgenic SOD1-G93A mice were intraperitoneally injected with SFI (40 ml/kg) three times a week from 87 days of age. Motor function, survival, pathological manifestations in the brain, and Nrf2 pathway-related assessments of the mice were performed.

**Results:** SFI treatment efficiently postponed the disease onset (*p* = 0.022) and extended the overall survival (*p* = 0.038) of the SOD1-G93A mice. Moreover, SFI significantly reduced motor neuron loss (*p* < 0.001) and astrocytic activation (*p* < 0.05) in the motor cortex of the brain of SOD1-G93A mice at 130 days of age. The protective effects of SFI in the SOD1-G93A mice were associated with decreasing the level of malondialdehyde (*p* < 0.05) and increasing the levels of superoxide dismutase (*p* < 0.05), Nrf2 (*p* < 0.05), heme oxygenase-1 (*p* < 0.05), and glutathione S-transferase (*p* < 0.05) in the SOD1-G93A mice.

**Conclusion:** The SFI treatment efficiently extended the overall survival and improved the pathological manifestations of the brain *via* alleviating the OS injury and activating the Nrf2 pathway in the animal model of ALS, which made SFI a potentially promising candidate for ALS treatment.

## 1 Introduction

Amyotrophic lateral sclerosis (ALS) is a fatal neurodegenerative disease and the most frequent motor neuron disorder in adults, characterized by progressive loss of the upper and lower motor neurons (MNs) in the motor cortex, brainstem, and anterior horn of the spinal cord, which leads to weakness and atrophy of voluntary muscles (Kiernan et al., 2011; Guttenplan et al., 2020; Oggiano et al., 2021). Clinically, patients with ALS manifest a gradual loss of the ability to walk, speak, and swallow, most of whom will die due to respiratory failure within 3–5 years after symptoms onset (Zoccolella et al., 2006; Palese et al., 2019). Currently, riluzole and edaravone have been approved by the United States Food and Drug Administration (US-FDA) for the treatment of ALS. However, riluzole extends the patients’ lifespan by only 2–3 months, and the exact clinical efficacy of edaravone on ALS still lacks evidence from large-scale multi-center clinical trials (Cai and Yang, 2019). Therefore, the development of new reagents is still of great significance in the management of patients with ALS.

Although the pathogenesis of ALS had not been clearly clarified, among the pathogenic mechanisms in ALS, oxidative stress (OS), resulting from excessive reactive oxygen species (ROS) production and inefficient antioxidant defense, is implicated in the loss of MNs contributing decisively to neurodegeneration in ALS and its animal model (Singh et al., 2019). Clinical studies have demonstrated that levels of malondialdehyde (MDA) and 8-hydroxyguanosine, two markers for OS injury, were significantly increased in the peripheral blood of ALS patients (Mendez and Sattler, 2015; Blasco et al., 2017). Moreover, a high level of ROS was shown in the central nervous system (CNS) regions of patients with ALS (Guo et al., 2013; Niedzielska et al., 2016). Therefore, antioxidant therapies may represent a promising strategy in ALS treatment. Notably, the nuclear factor-E2-related factor (Nrf2), a redox-sensitive transcription factor, inducing the expression of antioxidant enzymes genes, such as glutathione-S-transferase (GST) and Heme Oxygenase-1 (HO-1), has been widely accepted to have an essential role in protecting neurons from OS damage (Lee and Johnson, 2004). Correspondingly, a reduction in Nrf2 mRNA level was found in ALS patients’ tissues compared to that in controls, indicating that abnormality in this signaling may contribute to MNs degeneration in ALS (Buendia et al., 2016). A series of antioxidants with the ability of Nrf2 pathway activation, such as curcumin, resveratrol, and sulforaphane, were proven to delay disease progression in the murine model of ALS (Duan et al., 2010; Jiang et al., 2011; Mancuso et al., 2014). Particularly, dimethyl Fumarate, a well-known Nrf2 pathway activator, was used in a clinical trial for sporadic ALS treatment and observed to preserve the MN function and stabilize the respiratory function of patients through inducing Nrf2 activity (Vucic et al., 2020). Collectively, the Nrf2 pathway through mitigating OS injury may be a potential therapeutic target for designing therapies for ALS.

Shenqi Fuzheng Injection (SFI), concocted from two kinds of Chinese medicinal herbs, Radix Codonopsis (the root of *Codonopsis pilosula*; Chinese name: Dangshen) and Radix Astragali (the root of *Astragalus*; Chinese name: Huangqi), was approved by the State Food and Drug Administration of the People’s Republic of China primarily as an antitumor auxiliary injection to be manufactured and marketed in China in the year 1999 (Livzon Pharmaceutical Group Inc.; production batch number: Z19990065). In the past 20 years, there have been series of reports regarding the favorable efficacy of SFI in its clinical application for lung cancer and gastric cancer, and no serious side effects have been observed (Lv et al., 2015). Furthermore, SFI was approved by the US-FDA for a randomized, double-blind, phase I trial in the United States in 2018 (NCT number: NCT04026321) (Lv et al., 2015; Vucic et al., 2020). Additionally, SFI has been demonstrated to have potent anti-inflammation, antioxidative stress, and neuroprotection effects in various studies *in vivo* and *in vitro* (Lv et al., 2015; Cai et al., 2016; Zhu et al., 2019a). Moreover, pretreatment with astragaloside IV, an active component of Radix Astragali, to human umbilical vein endothelial cells exposed to ox-LDL significantly enhanced cell viability and suppressed apoptosis and ROS production by increasing the expressions of Nrf2 and HO-1 (Zhu et al., 2019b). *Codonopsis pilosula* polysaccharide, a bio-active component of Radix Codonopsis, protected the murine macrophages from H_2_O_2_-induced oxidative damage by reducing the reproductive OS damage related to the Nrf2 pathway (Qin et al., 2019). Moreover, early intervention with SFI contributed to protecting the neurons and decreasing the infarct volumes in aged rats with cerebral ischemia/reperfusion injury by raising the level of superoxide dismutase (SOD) activity and lowering the level of MDA (Cai et al., 2016). However, whether treatment with SFI could efficiently reduce OS damage and inhibit neurodegeneration in a well-established murine model of ALS—transgenic SOD1-G93A mice, which expresses large amounts of G93A, the mutant form of human SOD1, and develops adult-onset neurodegeneration of motor neurons and progressive motor deficits leading to paralysis (Gurney et al., 1994), and what the underlying mechanisms are mediating the action of SFI have not been explored. Therefore, in the present study, we aim to investigate the survival, motor function, pathological changes, and possible regulatory mechanism in transgenic SOD1-G93A mice with SFI treatment.

## 2 Materials and Methods

### 2.1 Animals

Transgenic SOD1-G93A mice used in the present study were produced by the *in vitro* fertilization technology (Shanghai Model Organisms Center, China). The sperm from the transgenic SOD1-G93A mice was obtained from The Jackson Laboratory in Bar Harbor, ME, United States (Jiang et al., 2016). All mice were genotyped by the polymerase chain reaction on DNA from tail biopsies, as previously reported (Wate et al., 2005). All procedures were approved by the Research Ethics Committee of the Institute of Basic Theory, China Academy of Traditional Chinese Medicine. In this study, only male SOD1-G93A mice were included to avoid estrogen interference (Solomonov et al., 2016). Non-transgenic male SOD1-G93A mice from the same litter were divided into the wild-type (WT) group. All mice were randomly assigned and divided according to different treatments as follows: 1) wild-type mice with saline treatment (WT + saline, *n* = 18); 2) wild-type mice with SFI treatment (WT + SFI, *n* = 18); 3) SOD1-G93A mice with saline treatment (SOD1+saline; *n* = 18); 4) SOD1-G93A mice with SFI treatment (SOD1+SFI, *n* = 18). All animals were housed one per cage in a 12 h light/dark cycle. Moreover, six mice in each group were randomly selected for the survival experiment, which was conducted to determine the effects of SFI on motor functions and survival of the mice, and 12 mice in each group were used for pathological and Nrf2 pathway-related assessments, which were performed to determine the effects of SFI on pathological manifestations and investigate the mechanisms mediating the action of SFI.

### 2.2 Motor Function Assessments

Researchers involved in the present study were divided into two groups, the observer group and injection group, who were completely double-blinded to the experimental protocol. Observers assessed the body weight, motor function, and neurological score (NS) of the mice in each group always between 10 am and 1 pm to avoid diurnal variations. The intraperitoneal injection was performed after recording the above items.

The motor function was evaluated by the rotarod test (RT) three times per week starting at 87 days of age on a rotarod apparatus (model LE8505; Panlab, Barcelona, Spain), as previously described (Peng et al., 2018). Mice were placed on a rotating rod (30 mm in diameter, 50 mm in length, accelerated gradually from 4 to 40 rpm over 180 s), and the time they maintained their balance on the rotating cylinder was measured (Filali et al., 2011). Each mouse was given three attempts, each mouse’s longest time maintained on the rod was recorded, and 180 s was chosen as the arbitrary maximum cut-off time. At 70 days of age, the mice firstly received an adaptation period of 10 days of daily practice on the rotarod.

The NS developed by the ALS Therapy Development Institute was from NS 0 to NS 4 (Hatzipetros et al., 2015), which was assessed three times per week starting at 87 days of age. 1) NS 0 (presymptomatic): pinching the tail of the mice and lifting it, the hindlimbs of the mice show a normal splay and stay for more than 2 s. The gait of the mice is normal. 2) NS 1 (first symptom or disease onset): suspending the mice by the tail, the hindlimb presents an abnormal splay. The gait of the mice is normal or a slightly slow gait. 3) NS 2 (onset of paresis): suspending the mice by the tail, the hindlimb is partially or completely collapsed, not extending much. The gait of the mice is abnormal and the hindlimb is used for forward motion; however, the toes curl downwards at least twice during a 90 cm walk or any part of the foot is dragging along the cage/table bottom. When the mouse is placed on its left and right side, it is able to right itself within 10 s from both sides. 4) NS 3 (paralysis): suspending the mice by the tail, there is rigid paralysis in the hindlimb or minimal joint movement. The gait of the mice is abnormal and there is forward motion; however, the hindlimb is not being used for forward motion. When the mouse is placed on its left and right side, it is able to right itself within 10 s from both sides. 5) NS 4 (end-point): when the mouse is placed on its left and right side, it is unable to right itself within 10 s from either side.

### 2.3 Treatment

The intraperitoneal injection dose of SFI was 40 ml/kg (250 ml of SFI contains 10 g of Radix Astragali and 10 g of Radix Codonopsis; Livzon Pharmaceutical Group Co., Ltd., Zhuhai, Guangdong Province, China; Batch No. Z19990065) (Cai et al., 2016). The injection was performed from 87 days of age three times a week until the mice reached NS 4 (survival experiment) and until 130 days of age (postsymptomatic stage) for mice in each group (pathological experiment). Mice in groups of WT + saline and SOD1 + saline were injected with an equal volume of saline.

### 2.4 Blood Sampling

Blood samples were collected at 130 days of age for mice in each group. Further, the blood was centrifuged at 4°C for 10 min at 3,000 rpm for serum and stored at −80°C until assayed.

### 2.5 Histology and Immunohistochemistry

At 130 days of age, mice in each group were subjected to perfusion with 4% (w/v) paraformaldehyde under deep anesthesia (10% chloralhydrate, 0.2 ml/mouse, intraperitoneally). Their brains were removed carefully and embedded in paraffin. Three brain sections (5 μm/each, interval of 100 μm) of individual mice were stained with hematoxylin & eosin (H&E) and Nissl for routine evaluation of neurons.

The levels of glial fibrillary acidic protein (GFAP), Nrf2, HO-1, and GST expression in the brain tissue were characterized by immunohistochemistry. Briefly, the brain tissue sections were deparaffinized, rehydrated, and treated with 3% (v/v) H_2_O_2_ in methanol for 30 min, followed by blocking with 5% (w/v) fat-free dry milk for 1 h. The sections were incubated with anti-GFAP (1:200, Bioworld Technology, Inc. United States), anti-Nrf2 (L593) (1:100, Bioworld Technology), anti-HO-1 (1:200, Bioworld Technology), or anti-GST (1:200, Proteintech Group, Rosemont, IL, United States) antibodies at 4°C overnight. After being washed, the bound antibodies were detected with biotinylated secondary antibodies and the ABC kit and visualized using diaminobenzidine, followed by examination under a light microscope (Olympus BX60, Japan). Histopathological images were analyzed using the Image-Pro Plus 6.0 software (Media Cybernetics, Inc., Rockville, MD, United States).

### 2.6 Western Blot

The brain tissues in the primary motor cortex (M1) region were dissected at 130 days of age and frozen immediately in liquid nitrogen. The total proteins were prepared using a protein extraction kit (Applygen Technologies, Beijing, China), according to the manufacturer’s instructions. The concentrations of proteins were measured using a BCA protein assay reagent kit (Novagen, Madison, WI, United States). Equal amounts of proteins were separated by sodium dodecyl sulfate–polyacrylamide gel electrophoresis (SDS-PAGE) and transferred onto polyvinylidene fluoride membranes (PVDF, Pierce Chemical, Rockford, IL, United States). Nonspecific binding sites were blocked with 5% skimmed milk in tris-buffered saline Tween (TBS-T) and then incubated overnight at 4°C with primary antibodies against Nrf2 (L593) (1:1,000, Bioworld Technology), HO-1 (1:1,000, Bioworld Technology), or control β-actin (1:2,000, Bioworld Technology), respectively. After being washed with TBS-T three times, the bound antibodies were detected with corresponding secondary antibodies (1:10,000, KPL, MD, United States). The relative levels of the target protein to control protein were quantified by enhanced chemiluminescence. Results were further assessed using the ImageJ 1.46 software (NIH, Bethesda, MD, United States) for density analysis, and the ratio of target protein expression/actin expression was also calculated.

### 2.7 ELISA Analysis

The concentrations of superoxide dismutase (SOD, Nanjing Jiancheng Bioengineering Institute, China) and MDA (Nanjing Jiancheng Bioengineering Institute) in the serum were determined by the commercially available ELISA kits following the manufacturer’s instructions.

### 2.8 Statistical Analysis

Data were expressed as mean ± standard error of the mean (SEM). ANOVA analysis followed by the least significant difference (LSD) test was used to assess differences among multiple groups, and the Mann–Whitney U test was used to evaluate differences between two groups. The time taken to reach a particular NS was analyzed using the Kaplan–Meier survival analysis with the Log-rank test for statistical significance. *p* < 0.05 was considered as statistical significance. IBM SPSS 21.0 software (IBM Japan, Tokyo, Japan) and GraphPad Prism7 software (MDF Co. Ltd., Tokyo, Japan) were applied for statistical analysis.

## 3 Results

### 3.1 Effects of Shenqi Fuzheng Injection on Weight, Disease Progression, and Motor Functions of Transgenic SOD1-G93A Mice

**FIGURE 1:**
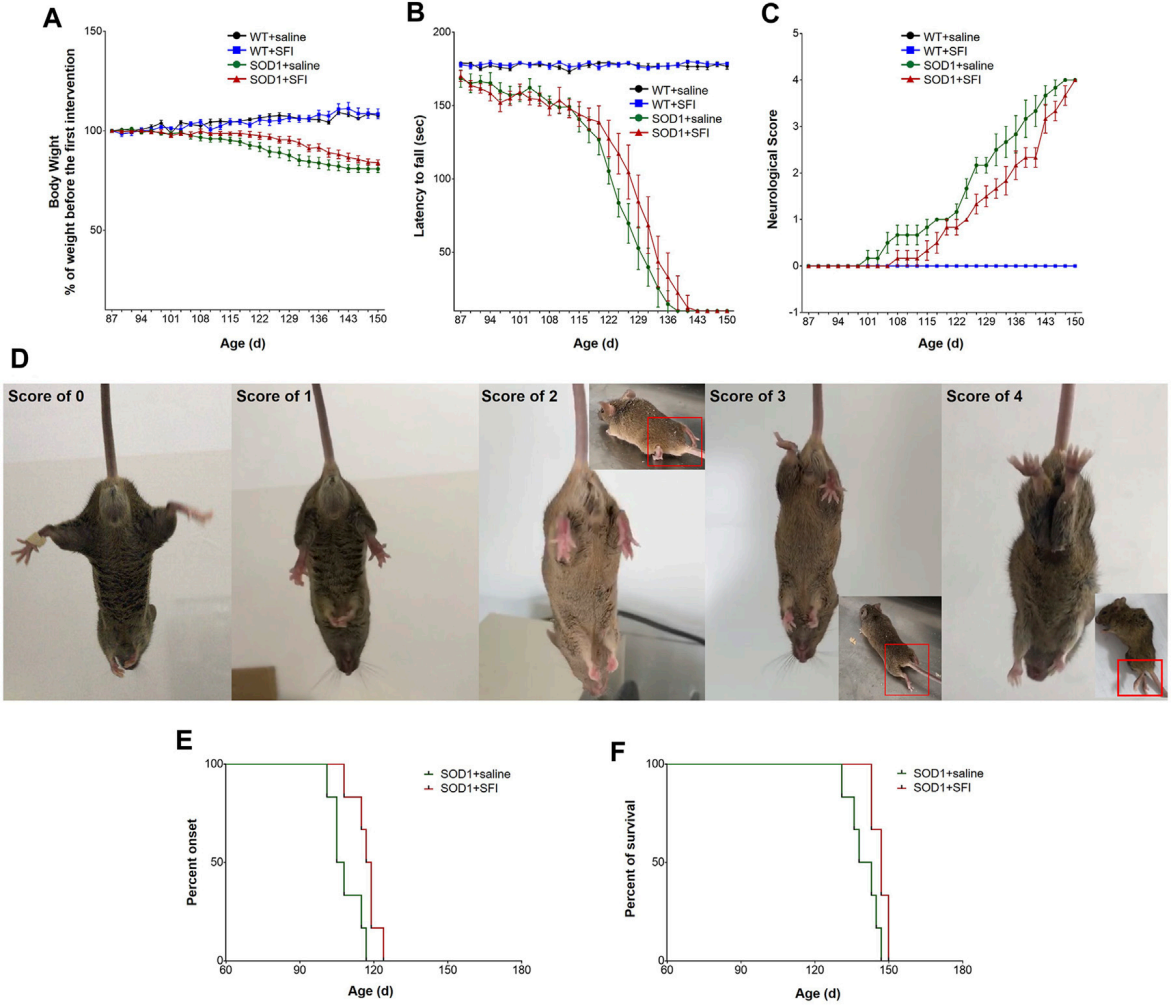
Shenqi Fuzheng Injection (SFI) treatment delayed the disease onset, extended the survival, and improved the motor performance of SOD1-G93A mice. Data are shown for the wild-type mice with saline treatment (WT + saline, *n* = 6), wild-type mice with SFI treatment (WT + SFI, *n* = 6), SOD1-G93A mice with saline treatment (SOD1+saline, *n* = 6), and SOD1-G93A mice with SFI treatment (SOD1+SFI, *n* = 6) and presented as mean ± standard error of mean. SFI treatment had no significant effects on the body weight in SOD1-G93A mice (**(A)**, *p* > 0.05). SFI treatment did not significantly improve the locomotion assessed using the rotarod test in SOD1-G93A mice (**(B)**, *p* > 0.05). SFI treatment did not significantly improve the neurological score (NS) in SOD1-G93A mice (**(C**), *p* > 0.05). The representative images of the different NS in SOD1-G93A mice are shown in **(D)**. SFI treatment delayed the disease onset (the time taken to reach NS 1) of mice in the group of SOD1+SFI compared to that in the group of SOD1+saline (**(E**), *p* = 0.022). SFI treatment extended the lifespan (the time taken to reach NS 4) of mice in the group of SOD1+SFI compared to that in the group of SOD1+saline (**(F)**, *p* = 0.038).

The body weight of mice in the groups of SOD1+SFI and SOD1+saline both started to decline from around 122 days of age. Moreover, there was no statistically significant difference in body weight between the SFI-treated mice (transgenic and WT) and saline-treated ones throughout the disease course, suggesting that SFI had little effect on the bodyweight of ALS model mice (Figure 1A).

Regarding the motor function evaluated by the RT, the rotating rod maintaining duration of mice in both the SOD1+SFI group and SOD1+saline groups dropped sharply from 115 days of age. Furthermore, at 131 days old, the RT could not be completed by mice in both groups. Regretfully, throughout the disease course, the RT performance of mice in the SOD1+SFI group was indistinguishable from that of mice in the SOD1+saline group (Figure 1B).

SFI treatment delayed the disease onset (the time taken to reach NS 1) by 8.5 days on average (SOD1+saline group, 108.5 ± 2.6 days; SOD1+SFI group, 117.0 ± 2.2 days; *p* = 0.022). The time for the SOD1+SFI group to reach NS 2 was postponed by 6.7 days on average compared with that for the SOD1+saline group (SOD1+saline group, 124.3 ± 0.6 days; SOD1+SFI group, 131.0 ± 2.1 days; *p* = 0.006). The time required for the SOD1+SFI group to reach NS 3 was delayed by 5.3 days on average (SOD1+saline group, 135.2 ± 2.6 days; SOD1+SFI group, 140.5 ± 2.0 days; *p* = 0.084). SFI treatment extended the lifespan (the time taken to reach NS 4) of mice in the SOD1+SFI group by 6.7 days on average (SOD1+saline group, 140.0 ± 2.5 days; SOD1+SFI group, 146.7 ± 1.3 days; *p* = 0.038). In contrast, SFI treatment did not prolong the duration between the first symptom (NS 1) and the end-point (NS 4) (SOD1+saline group, 31.5 ± 0.8 days; SOD1+SFI group, 29.7 ± 2.8 days; *p* = 0.720). (Figures 1C–F; Table 1).

**TABLE 1:** The time taken to reach different neurological scores (NS) in the transgenic SOD1-G93A mice.

	SOD1+saline *n* = 6	SOD1+SFI *n* = 6	*p* value
NS 1 (d), mean ± SEM	108.5 ± 2.6	117.0 ± 2.2	**0.022**
NS 2 (d), mean ± SEM	124.3 ± 0.6	131.0 ± 2.1	**0.006**
NS 3 (d), mean ± SEM	135.2 ± 2.6	140.5 ± 2.0	0.084
NS 4 (d), mean ± SEM	140.0 ± 2.5	146.7 ± 1.3	**0.038**
From NS 1 to NS 4 (d), mean ± SEM	31.5 ± 0.8	29.7 ± 2.8	0.720

d, days; SEM, standard error of the mean; SFI, Shenqi Fuzheng Injection; SOD1, superoxide dismutase 1; SOD1+saline, SOD1-G93A mice with saline treatment; SOD1+SFI, SOD1-G93A mice with SFI treatment. Bold type indicates a significant difference.

Additionally, among WT mice, there was no obvious difference in the body weight, motor function, and lifespan between those with SFI treatment and with saline treatment.

### 3.2 Effects of Shenqi Fuzheng Injection on MNs Loss and Astrocytic Activation in the M1 Region of the Brain of Transgenic SOD1-G93A Mice

**FIGURE 2:**
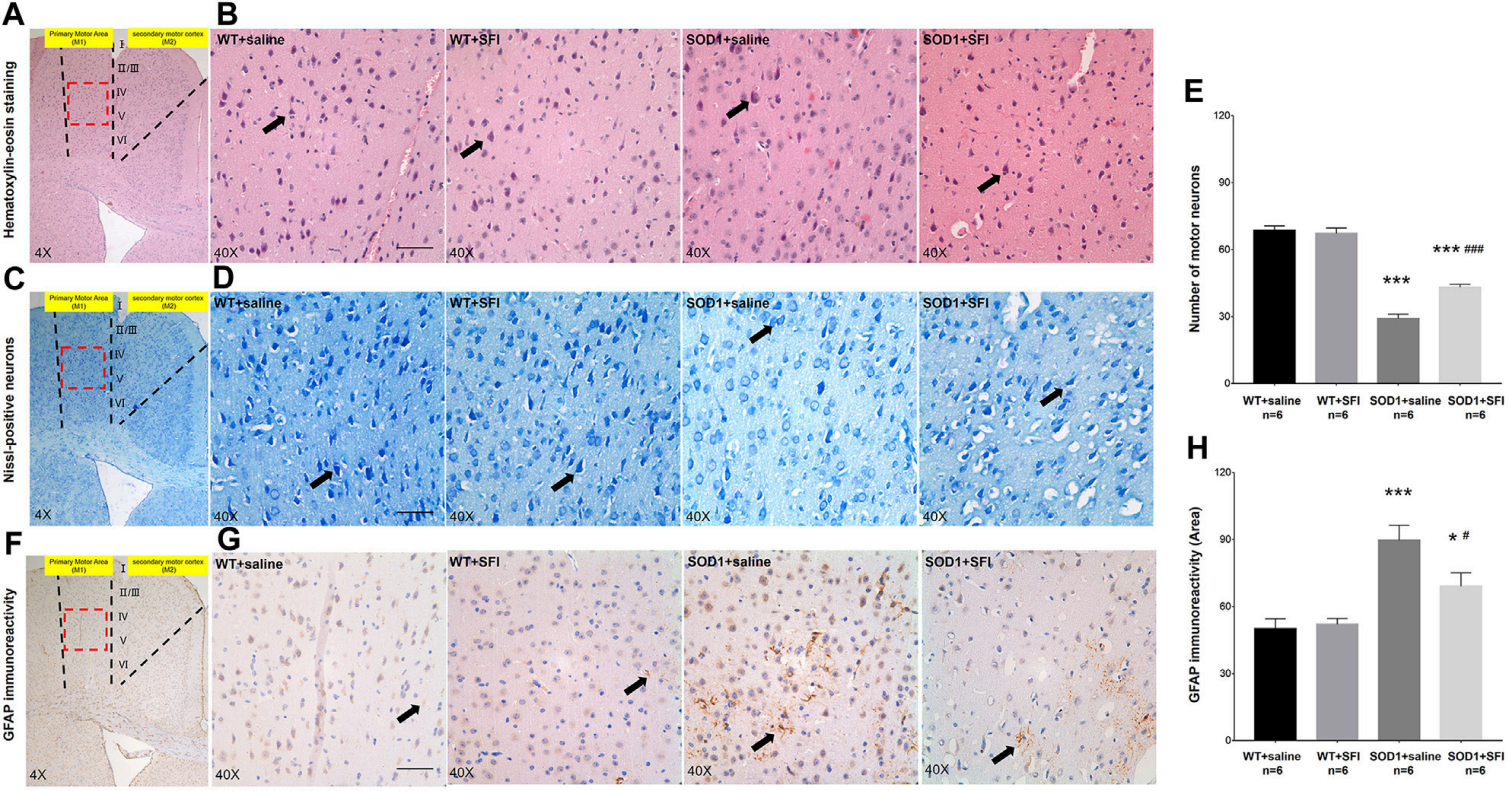
Shenqi Fuzheng Injection (SFI) treatment reduced the motor neuron loss and astrocytic activation in the brain of SOD1-G93A mice. Data shown are representative images and expressed as mean ± standard error of mean in the wild-type mice with saline treatment (WT + saline, *n* = 6), wild-type mice with SFI treatment (WT + SFI, *n* = 6), SOD1-G93A mice with saline treatment (SOD1+saline, *n* = 6), and SOD1-G93A mice with SFI treatment (SOD1+SFI, *n* = 6). SFI treatment increased the number of motor neurons indicated by HE staining **(A,B)** and Nissl staining **(C,D)** in the motor cortex of the brain (black arrows indicated motor neurons; scale bar = 50 μm). The quantification of motor neurons in the motor cortex of the brain **(E)**. SFI treatment reduced the astrocytic activation indicated by the GFAP staining in the motor cortex of the brain (**(F,G)** black arrows indicated GFAP-positive cells; scale bar = 50 μm). The quantification of astrocytic activation in the motor cortex of the brain **(H)**. The red dotted lines region in the images of **(A, C, F)** was magnified 10× and shown in the images of **(B, D, G)**, respectively. *p < 0.05 or ***p < 0.001 vs. the WT + saline group; ^#^ *p* < 0.05 or ^###^ *p* < 0.001 vs. the SOD1+saline group.

The pathological manifestations in the M1 region of the brain at 130 days of age were quantified by HE, Nissl, and immunohistochemistry staining (Clark et al., 2017). There were plenty of MNs with long and thin synapses, full cell bodies, and deep staining of nuclei in the M1 region of mice in groups of WT + SFI and WT + saline. In contrast, there were sharply decreased numbers of MNs with fuzzy cell bodies and staining in the M1 region of mice in the SOD1+saline group compared to those in the group of WT + saline (*p* < 0.001). On the other hand, obviously increased numbers of MNs with comparative normal morphology and staining in the M1 region of the brain were observed in the SOD1+SFI mice compared with those in the SOD1+saline mice (*p* < 0.05) (Figures 2A–E).

Astrocytic activation was assessed in the M1 region of the brain at 130 days by the immunohistochemistry staining of GFAP. The results showed that there were few GFAP-positive glial cells in both the WT+SFI group and WT + saline group. On the other hand, obviously increased GFAP-positive glial cells were observed in the SOD1+saline group compared to those in the WT + saline group (*p* < 0.001). Furthermore, there were significantly fewer GFAP-positive glial cells in the SOD1+ SFI group than those in the SOD1+saline group (*p* < 0.05). (Figures 2F–H).

### 3.3 Effects of Shenqi Fuzheng Injection on Oxidative Stress and Nrf2 Pathway in the M1 Region of the Brain of Transgenic SOD1-G93A Mice

**FIGURE 3:**
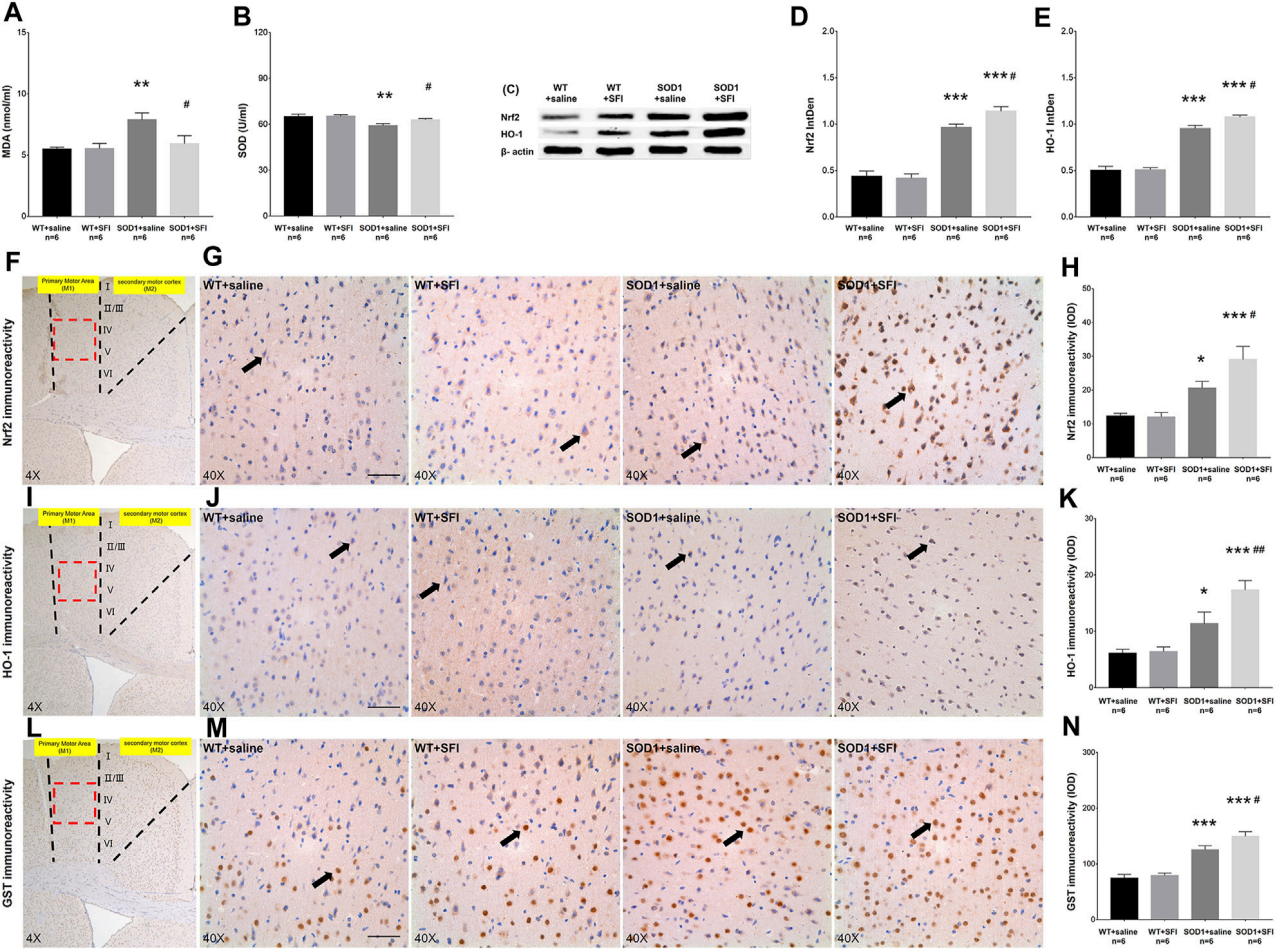
Shenqi Fuzheng Injection (SFI) treatment alleviated the oxidative stress in the serum and brain of SOD1-G93A mice. Data shown are representative images and expressed as mean ± standard error of mean in the wild-type mice with saline treatment (WT + saline, *n* = 6), wild-type mice with SFI treatment (WT + SFI, *n* = 6), SOD1-G93A mice with saline treatment (SOD1+saline, *n* = 6), and SOD1-G93A mice with SFI treatment (SOD1+SFI, *n* = 6). The levels of malondialdehyde (MDA) in the serum **(A)**. The levels of superoxide dismutase (SOD) in the serum **(B)**. Western blot analysis of the Nrf2 and HO-1 expression in the brain of individual mice **(C–E)**. β-actin was used as the internal standard. Immunohistochemistry analysis of the relative levels of Nrf2 (**(F–H)** black arrows indicate Nrf2 positive cells), HO-1 (**(I–K)** black arrows indicate HO-1 positive cells), and GST (**(L–N)** black arrows indicate GST positive cells) in the brain of individual mice (scale bar = 50 μm). The red dotted lines region in the images of **(F, I, L**) was magnified 10× and shown in the images of **(G, J, M**), respectively. *p < 0.05 or **p < 0.01 or ***p < 0.001 vs. the WT + saline group; ^#^ *p* < 0.05 or ^##^ *p* < 0.01 vs. the SOD1+saline group.

OS has been proven to play a key role in the pathogenesis of ALS. We measured the levels of MDA, a marker of lipid peroxidation, in the serum of mice at 130 days old in the different groups. In comparison with those in the WT mice treated with saline, significantly higher levels of MDA were detected in the serum of the mice in the SOD1+saline group (*p* < 0.01). In contrast, in comparison with those in the SOD1+saline group, significantly lower levels of MDA were observed in the serum of the mice in the SOD1+SFI group (*p* < 0.05) (Figure 3A). Besides, the concentrations of SOD, an antioxidant enzyme in the serum, significantly decreased in the SOD1+saline group compared with those in the WT + saline group (*p* < 0.01) and SOD1+SFI group (*p* < 0.05) (Figure 3B).

It is widely accepted that the Nrf2 pathway and related antioxidant enzymes, such as HO-1, GST, and SOD, are critical mediators of OS. We further examined the effects of SFI treatment on the levels of Nrf2, HO-1, and GST in the M1 region of the brain and the SOD expression in the serum of mice in different groups at 130 days of age using the methods of immunohistochemistry, Western blot, and ELISA. The levels of Nrf2 (*p* < 0.05), HO-1 (*p* < 0.05), and GST (*p* < 0.001) were higher in the SOD1+saline group than those in the WT + saline group. In contrast, the levels of Nrf2 (*p* < 0.05), HO-1 (*p* < 0.01), and GST (*p* < 0.05) increased significantly in the SOD1+SFI group compared to those in the SOD1+saline group (Figures 3F–N). A similar pattern of Nrf2 and HO-1 expression was detected in the brain of mice in different groups by the Western blot assays (Figures 3C–E). Taken together, treatment with SFI enhanced the Nrf2 expression and activation, which resulted in upregulating the expression of various antioxidant enzymes in the peripheral blood and CNS and contributing to its antioxidant ability in ALS model mice.

## 4 Discussion

In the present study, we investigated the effects of SFI, an injection concocted from Chinese medicinal herbs, in a well-established animal model of ALS, transgenic SOD1-G93A mice, and revealed that SFI significantly postponed the disease onset and extended the survival in the SOD1-G93A mice. Although SFI did not evidently reduce the motor function impairment of the SOD1-G93A mice, it efficiently improved the pathological manifestations by reducing the MNs loss and astrocytic activation in the brain via activating the Nrf2 pathway, inducing antioxidant enzymes such as HO-1, GST, and SOD expression, and thereby alleviating the OS damage in both CNS and peripheral nervous system.

The significant role of OS in the pathogenesis of ALS and its animal model has been established for years, on the basis of which inducing sufficient antioxidant production and improving the body’s antioxidative stress ability were believed to be a promising target for ALS management (Guo et al., 2013; Niedzielska et al., 2016; Singh et al., 2019). Among the numerous candidates for antioxidant induction, the Nrf2 pathway is proven to be critical in cellular defense against OS in the liver, lung, and brain (Chen et al., 2006; Innamorato et al., 2008; Osburn et al., 2008). Activation of the Nrf2 pathway could exert powerful antioxidant and anti-inflammatory effects and play an important neuroprotective role in ALS, multiple sclerosis, Alzheimer’s disease, Parkinson’s syndrome, and other neurodegenerative disorders (Jiang et al., 2011; Mancuso et al., 2014; Fão et al., 2019; Michaličková et al., 2020; Osama et al., 2020). Although a variety of natural extracts for Nrf2 pathway activation have been proven to be effective in inhibiting neurodegeneration, slowing disease progression, and prolonging survival in ALS animal experiments, potential limitations still inhibited their further clinical transformation, such as poor efficacy in clinical trials and certain toxicity of themselves (Andrews et al., 2019). Therefore, the development of novel Nrf2 pathway activators with non-toxicity, especially in-depth exploration of the potential Nrf2 activation mechanisms for existing clinical medication, and thereby expansion of their clinical application scope or diseases will be of great value for ALS treatment.

SFI has been marketed as an antitumor auxiliary medicine as early as in the year 1999 in China and approved for clinical trials in the United States in 2018, which guaranteed its safety in clinical application. Previous studies have also revealed the potential roles of antioxidative stress and Nrf2 pathway activation of SFI and its active component in stroke and other neurological disorders (Cai et al., 2016; Zhu et al., 2019b; Qin et al., 2019). Consistent with those findings, in the present study, we observed that SFI could efficiently induce antioxidant enzymes such as HO-1, GST, and SOD expression in both CNS and peripheral blood and alleviate the OS injury of the SOD1-G93A mice. Due to the above effects, SFI treatment evidently reduced MNs loss and astrocytic activation, the typical pathological characteristics of ALS, in the brain of the SOD1-G93A mice. Although SFI application could not significantly improve the motor function impairment throughout the disease course, which may be partly due to the limited number of experimental animals involved in this study, the findings that SFI could extend the lifespan, improve the pathological manifestations, and activate the Nrf2 pathway still made us recognize the possibility of SFI as a potential treatment strategy for ALS and encouraged us to make further investigations.

A few limitations are worthy of being particularly mentioned and should be explored in future studies. First, the number of experimental animals was insufficient, especially for mice involved in the survival and motor function assessment experiment. Second, RT was the only measurement for motor function and other behavioral tests involving hind paw footprint and grip test are lacking, which made us unable to evaluate the motor function of the mice comprehensively. Third, although we observed that SFI could significantly delay the disease onset of the SOD1-G93A mice, the related pathological test at that stage was not conducted correspondingly. Moreover, the relevant assessments of the Nrf2 pathway activation from the perspective of gene expression were lacking. Fourth, the present study focused on the motor cortex of the brain for the pathological and Nrf2 pathway assessment, and the corresponding evaluations in other regions of the CNS, including the spinal cord or medulla, were lacking. Therefore, further studies with more experimental animals involved in both the survival experiment and pathological tests, comprehensive application of multiple behavioral testing methods, and more precise and detailed pathological and Nrf2 pathway assessments in multiple regions of the CNS in both presymptomatic and postsymptomatic stages of the disease will be needed to confirm the findings of the current study and further investigate the effects and possible underlying mechanisms of SFI in an ALS animal model in depth.

## 5 Conclusion

The SFI treatment efficiently extended the overall survival and improved the pathological manifestations of the brain by alleviating OS injury and activating the Nrf2 pathway in the animal model of ALS, which made SFI a potentially promising candidate for ALS management.

## Data Availability

The original contributions presented in the study are included in the article/supplementary material; further inquiries can be directed to the corresponding author.
